# Innate response to Foamy viruses

**DOI:** 10.1186/1742-4690-8-S1-A239

**Published:** 2011-06-06

**Authors:** Réjane Rua, Alice Lepelley, Antoine Gessain, Olivier Schwartz

**Affiliations:** 1 Epidemiology and Physiopathology of Oncogenic Retroviruses Unit, Pasteur Institute, Paris, 75015, France; 2 Virus and Immunity Unit, Pasteur Institute, Paris, 75015, France

## Background

Foamy viruses (FV) are non-pathogenic retroviruses widespread in various monkey species. FV can be transmitted through bites from monkeys to humans, in which viral loads remain low. No secondary human transmission has been reported. Little is known about the ability of FV to trigger an innate immune response. Few reports suggested that FV does not induce type-I interferon (IFN) in cultures of non-hematopoietic cells.

## Materials and methods

We have examined how FV particles and FV-infected cells are sensed by human hematopoietic cells, with a focus on plasmacytoid dendritic cells (pDCs), the main type-I IFN producing cells.

## Results

A human pDC-like cell line (Gen2.2), as well as primary pDCs and PBMCs respond to FV by producing high levels of IFN-I and by expressing the interferon-stimulated gene MxA. This response is rapid (12h). Less than 100 FV infected cells are sufficient to trigger an IFN response. IFN release is blocked by an inhibitor of endosomal acidification (Bafilomycin A1) and by a TLR-7 /9 antagonist (A151). Silencing experiments in Gen2.2 further demonstrated that TLR-7 is involved in FV recognition.

## Conclusions

FV is an efficient inducer of type-I IFN by pDCs and by PBMCs. This induction is predominantly mediated by TLR-7 in pDCs. We are currently characterizing further FV sensing pathways in other cell types and determining the role of viral proteins and nucleic acids in this recognition. This previously underestimated activation of the innate immune response by FV is likely involved in the non-pathogenicity of FV in humans.

